# Promotora intervention for metabolic and mental health to reduce type 2 diabetes risk: a pilot randomized controlled trial

**DOI:** 10.1038/s41598-024-83482-5

**Published:** 2024-12-30

**Authors:** Maria C. Vargas, Joshua B. Katz, Azucena Lopez, Arturo Carrillo, Dyanna L. Gregory, Matthew J. O’Brien

**Affiliations:** 1 Institute of Public Health and Medicine, Northwestern University Feinberg School of Medicine, Chicago, USA; 2 Division of General Internal Medicine and Geriatrics, Department of Medicine, Northwestern University Feinberg School of Medicine, Chicago, USA; 3 Institute for Policy Research, Northwestern University, Evanston, USA; 4 Division of Digestive & Liver Diseases, Metabolism and Molecular Medicine, The University of Texas Southwestern Medical Center, Dallas, USA; 5 Brighton Park Neighborhood Council, Chicago, USA; 6 Division of Endocrinology, Diabetes, and Metabolism, University of Illinois College of Medicine at Chicago, Chicago, USA; 7 Division of General Internal Medicine and Geriatrics, Northwestern University, 750 N. Lake Shore Drive, 10th Floor, Chicago, IL 60611 USA

**Keywords:** Diabetes prevention, Psychological stress, Promotoras, Community health workers, Hispanic health, Human behaviour, Pre-diabetes, Translational research

## Abstract

The objective of this study was to develop and evaluate a novel behavioral intervention (PRIME2) that integrates evidence-based approaches for reducing diabetes risk and perceived stress. We conducted a pilot randomized controlled trial of the 16-session PRIME2 intervention vs. usual care among 40 Spanish-speaking Latinx adults with prediabetes and body mass index (BMI) ≥ 25 kg/m^2^. The 3-month change in weight and perceived stress were co-primary outcomes. Secondary outcomes were 3-month changes in hemoglobin A1c (HbA1c), blood pressure, and depressive symptoms. Study participants were women (95%) with mean age 49.9 (± 9.3) years, moderate levels of perceived stress, and risk factors for diabetes, including mean BMI 33.3 (± 5.4) kg/m^2^ and elevated HbA1c [mean 6.0% (± 0.2)]. PRIME2 participants demonstrated significantly greater 3-month weight loss relative to usual care (− 3.7lbs, *p* = 0.02). Reductions in perceived stress in both study arms were similar. There were no significant differences in secondary outcomes among PRIME2 participants vs. usual care. This pilot trial demonstrates the feasibility and preliminary weight loss effectiveness of the novel PRIME2 intervention, without observing a significant difference in perceived stress between study arms. Future research should include larger randomized samples that enable a definitive evaluation of intervention effects on both metabolic and mental health endpoints.

**Supplementary Information:**

The online version contains supplementary material available at 10.1038/s41598-024-83482-5.

## Background

Prediabetes affects 98 million U.S. adults, representing approximately one-third of the adult population^1^. While the prevalence of prediabetes is similar across racial/ethnic groups^2^, large disparities exist in the burden of diabetes by race/ethnicity. U.S. Hispanic/Latino adults experience the greatest burden of diabetes with an age- and sex-adjusted prevalence of 22.1%, relative to 12.1% and 20.4% among white and Black adults, respectively^3^. U.S. Hispanics/Latinos also have high rates of undiagnosed diabetes, accounting for approximately half of diabetes cases in this group^2^. These data highlight the urgent importance of diabetes prevention efforts in Hispanic/Latino communities, which have the potential to reduce observed disparities.

Prior research has identified an association between psychological stress and an increased risk of developing diabetes across population groups, including Hispanics/Latinos^4–8^. The link between stress and diabetes is explained, in part, by common underlying pathophysiologic mechanisms (e.g. upregulation of inflammatory markers and cortisol release), as well as shared behavioral correlates (e.g. unhealthy diets, physical inactivity, and inadequate sleep)^9,10^. These observations suggest that promoting healthy lifestyle behaviors may help lower diabetes risk, and lessen the impact of psychological stress on developing diabetes^6^.

Landmark clinical trials of adults with prediabetes, representing all racial/ethnic backgrounds, have established that intensive lifestyle interventions can effectively prevent or delay diabetes^11–14^. For example, the U.S. Diabetes Prevention Program demonstrated a 58% reduction in diabetes incidence with a structured lifestyle intervention focused on achieving 150 min of physical activity weekly and 7% weight loss from baseline^11^. Many translational trials have studied this group-based intervention, reporting weight loss comparable to that observed in the original efficacy trial^15,16^. One of these examined outcomes according to participants’ level of perceived stress. This study found lower adherence to lifestyle goals and less weight loss among participants with elevated stress levels, relative to those with low perceived stress (− 7.2% from baseline vs. − 2.9%, *p* < 0.001)^17^. The links observed between perceived stress and glycemic control from prior basic, epidemiologic and intervention research, suggest the potential for addressing healthy lifestyle change and stress reduction using an integrated approach.

The objective of the current study was to develop and evaluate a novel pilot intervention integrating evidence-based stress reduction approaches into the Diabetes Prevention Program lifestyle program. Our intervention was delivered by community health workers (hereafter “promotoras”), whom prior research has identified as an effective lay workforce for delivering behavioral interventions including those focused on diabetes prevention^15^. The current paper describes the development of this intervention, as well as a pilot randomized controlled trial that examined its feasibility and preliminary effectiveness.

## Methods

### Study design

We conducted a pilot trial of the novel Promotora Intervention for Metabolic and Mental Health (PRIME2) that parallel-randomized participants in a 1:1 ratio to receive the PRIME2 intervention or enhanced usual care. The random allocation sequence was generated independently by a statistician and made available only to the research coordinator, who ultimately assigned participants to the study interventions. A secure, web-based application hosted at Northwestern University was used to implement the allocation sequence^18^. The nature of the study design precluded blinding participants or promotoras to treatment assignments. The study protocol was approved by the Northwestern University Institutional Review Board (Reference# STU00202244) and was first recorded in the National Clinical Trials Registry (NCT# NCT03372018) on 13/12/2017. The study was designed following the Consolidated Standards of Reporting Trials (CONSORT) and conducted in accordance with the Declaration of Helsinki^19,20^.

### Participants and setting

The inclusion criteria were: (1) Latinx ethnicity; (2) Spanish language fluency; (3) age ≥ 18 years; (4) prediabetes with hemoglobin A1c (HbA1c) of 5.7-6.4%; and (5) BMI ≥ 25 kg/m2. Given the high prevalence of psychosocial stress observed in the target community by collaborating organizations, baseline stress levels were not assessed as part of the study eligibility criteria. The exclusion criteria were: (1) current or planned pregnancy during the study period; (2) self-reported chronic conditions that could influence weight (e.g. uncontrolled thyroid disease, cancer, and HIV) or affect potential participants’ ability to participate (e.g. severe osteoarthritis); 6) self-reported medications that could affect weight or glucose metabolism (e.g. phentermine, topiramate, bupropion, and systemic glucocorticoids); and 5) measured blood pressure > 180/105 mmHg. Eligibility criteria were assessed using questionnaires and biomarkers collected during screening study visits. The pilot study included 40 participants, 20 in each arm. Given the goals of this pilot study to evaluate the feasibility and preliminary effectiveness of the novel PRIME2 intervention, we did not include a priori power calculations based on different interventions that were previously published in the literature. This decision was further justified by findings from the landmark Diabetes Prevention Program that 1 kg of weight loss resulted in a 16% reduction in diabetes incidence^21^. Therefore, 1 kg of weight loss was considered the minimal clinically important difference^22^.

The PRIME2 pilot trial was conducted in partnership with three community-based organizations serving Latinx communities in Chicago. Recruitment methods included flyers, community events, and referrals from staff at the collaborating organizations. Potential participants were referred to the research coordinator, who administered a screening questionnaire, checked blood pressure, and collected a capillary blood sample for HbA1c assessment. Blood pressure was measured using an Arm Blood Pressure Monitor (Digital Automatic Blood Pressure Monitor HEM-907XL, Omron Healthcare Co Ltd) automated sphygmomanometer and HbA1c was assessed using the DCA Vantage 2000/Vantage portable bench-top analyzer. The same devices were used for assessment of cardiometabolic outcomes among study participants. Prior to enrollment, participants gave written informed consent.

### Study interventions

#### PRIME2 intervention

**Fig. 1:**
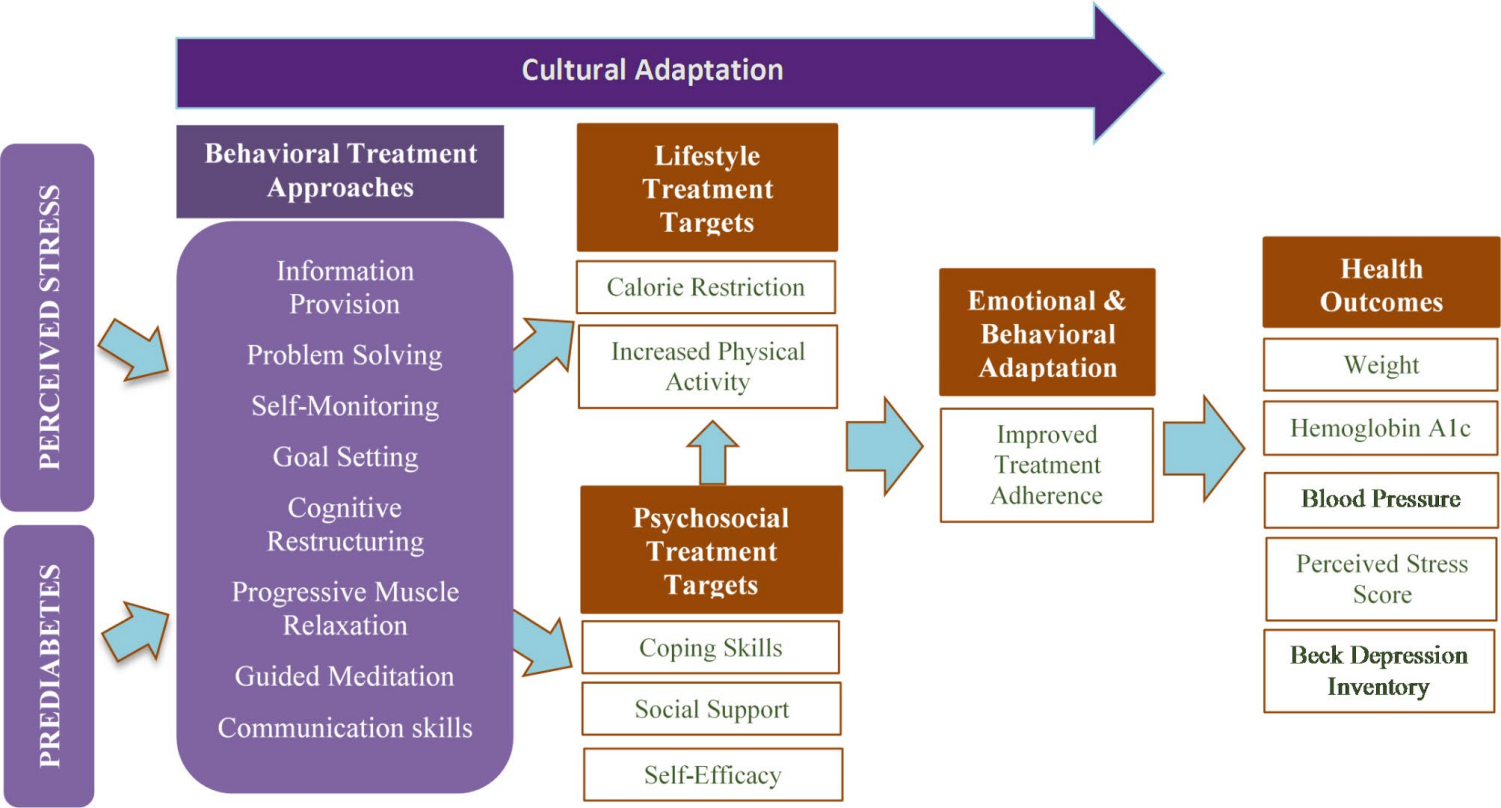
PRIME2 Conceptual Framework.

The PRIME2 intervention represents an integration of two evidence-based curricula targeting metabolic health (i.e. diabetes prevention) and mental health (i.e. perceived stress), both of which were studied previously in Spanish. This intervention was delivered at the collaborating community-based organizations by staff promotoras, who offer cultural humility and knowledge of local resources when providing health education^23^. In PRIME2, the promotoras delivered psychoeducation about core cognitive-behavioral concepts rather than provide group-based psychotherapy. The metabolic health content included in PRIME2 was adapted from the Diabetes Prevention Program, which has demonstrated effectiveness in diverse settings^15^, including trials conducted by the study team using promotoras^24–26^. This group-based intensive lifestyle program was designed to help participants lose weight by reducing overall caloric intake and promoting regular physical activity^27^. These behaviors are tracked weekly during the intervention using self-monitoring logs^27^. The mental health content in the PRIME2 intervention was adapted from the Cognitive-Behavioral Stress Management for Prostate Cancer Recovery (C-CBSM)^28^. This intervention focuses on the following evidence-based approaches for stress reduction: self-monitoring thoughts and stress levels, rational thought replacement, progressive muscle relaxation, guided meditation, and communication skills. The conceptual framework for the PRIME2 intervention (Fig. 1) demonstrates interconnections between prediabetes and perceived stress, including behavioral treatment approaches, treatment targets, and health outcomes related to both conditions.

Study investigators (MJO, MCV, AC) met weekly for 6 months to integrate selected content from these two sources into the 16-week PRIME2 intervention. All PRIME2 intervention materials were developed in Spanish by our fully bilingual team, using previously described methods and Spanish-language versions of publicly available original source material^25,29^. The PRIME2 curriculum consists of an integrated group-based DPP intervention with psychoeducation, guided throughout by cognitive behavioral therapy (Supplementary Table S1). Investigators reviewed all session materials with the promotoras who would ultimately deliver the intervention during 16 weekly sessions. These promotoras ensured cultural salience of the intervention materials for the target population by actively participating in curricular development process. Promotoras reviewed the PRIME2 curriculum for social and cultural appropriateness of targeted mental and metabolic health messages, making adaptations to promote their acceptance and effectiveness for the study population. Using promotoras to deliver the intervention, who were drawn from the same community as the participants, also fostered cultural salience and appropriateness throughout the study implementation. Upon completing the PRIME2 intervention materials, the promotoras were trained by delivering the sessions to the investigators, who provided real-time feedback. The study intervention was delivered from November/2018 to March/2019.

### Enhanced usual care intervention

Participants randomized to enhanced usual care attended six group educational sessions focused on diabetes. During these sessions, the research coordinator reviewed standardized educational materials about diabetes from the National Institute of Diabetes and Digestive and Kidney Disease^30^. Participants were encouraged to speak to their healthcare providers about management of their diabetes risk, which was not addressed during these sessions.

### Outcome measures

Data on metabolic and mental health outcomes were collected during baseline, 3-month, and 6-month assessments. Participant’s body weight was measured to the nearest 0.1 kg using a Seca 703 scale. Height was measured to the nearest 1 mm using a Seca stadiometer, and was used to calculate body mass index (BMI). Waist circumference was assessed using a measuring tape held around the top of the iliac crests at end-expiration. Participants’ blood pressure was measured using an Omron HEM-907XL sphygmomanometer after being seated for 15 min. Reported values represent the average of three consecutive blood pressure measurements. HbA1c values were obtained from a fingerstick capillary blood sample and analyzed using a DCA Vantage 2000 benchtop analyzer.

Mental health outcomes were assessed using questionnaires administered to participants in Spanish using previously validated translations of the following instruments. Perceived stress was assessed using Cohen’s 14-item Perceived Stress Scale, which has been widely used (α = 85)^31^. The 21-item Beck Depression inventory was used to measure participants’ depressive symptoms. This scale has also been validated in diverse populations (α = 0.91)^32^. Participant attendance served as the primary indicator of intervention feasibility. This outcome was measured as a continuous mean of sessions attended, as well as a dichotomous variable describing the proportion of participants who attended no sessions and ≥ 9 sessions. The latter represents an attendance threshold that is associated with clinically meaningful weight loss^33^.

The 3-month changes in body weight and perceived stress were considered the co-primary outcomes. To examine the durability of changes in metabolic and mental health outcomes from the PRIME2 intervention, exploratory 6-month data were collected on participants randomized to this group.

### Statistical analysis

Descriptive analyses were used to characterize the cohort with respect to sociodemographic characteristics, cardiometabolic outcomes, and mental health outcomes at baseline (Table 1). Differences between participants in the PRIME2 and enhanced usual care groups were assessed using Fisher’s exact tests for categorical variables and *t*-tests for continuous variables. Attendance at intervention sessions was analyzed descriptively, and its association with study outcomes was examined using independent *t*-tests. Analysis of 3-month outcomes followed an intention-to-treat principle where participants who completed data collection were analyzed according to their randomized group assignment, regardless of their level of adherence. The significance of differences in metabolic and mental health outcomes was examined using independent *t*-tests (Table 2). Exploratory 6-month data on participants randomized to the PRIME2 arm were analyzed using Wilcoxon signed-rank tests (Table 3). All analyses were conducted using StataSE v. 15 (College Station, TX).

**Table 1:** Baseline characteristics of PRIME2 participants by treatment assignment data are presented as means with standard deviation (± SD) or n (%).

Characteristic	Overall (*n* = 40)	PRIME2 (*n* = 20)	Standard care (*n* = 20)	*P* value
Sex, female	38 (95.0)	19 (95.0)	19 (95.0)	1.0
Age, years	49.9 ± 9.3	49.5 ± 9.5	50.4 ± 9.4	0.78
Education, years	9.3 ± 4.0	8.6 ± 3.9	10.1 ± 4.1	0.23
Household income, dollars	25,729.6 ± 12,366.8	25,000.0 ± 12,492.8	26,420.8 ± 12,547.4	0.73
Foreign born, n (%)	36 (90.0)	19 (95.0)	17 (85.0)	0.61
Family history of diabetes, n (%)	30 (75.0)	14 (70.0)	16 (80.0)	0.72
History of gestational diabetes, n (%)	5 (12.5)	3 (15)	2 (10.0)	1.00
Weight, lbs	179.7 ± 28.6	182.0 ± 28.0	177.4 ± 29.7	0.62
Body mass index, kg/m^2^	33.3 ± 5.4	33.6 ± 4.5	33.1 ± 6.3	0.80
Waist circumference, cm	102.4 ± 13.2	103.6 ± 11.0	101.3 ± 15.3	0.59
Hemoglobin A_1c_, %	6.0 ± 0.2	5.9 ± 0.2	6.0 ± 0.2	0.15
Systolic blood pressure, mmHg	117.1 ± 18.5	116.6 ± 15.6	117.6 ± 21.4	0.87
Diastolic blood pressure, mmHg	73.2 ± 9.8	72.3 ± 9.3	74.2 ± 10.5	0.56
Beck depression	11.3 ± 11.7	11.1 ± 9.6	11.5 ± 13.7	0.92
Cohen’s stress	18.8 ± 2.8	19.0 ± 2.4	18.7 ± 3.2	0.70

**Table 2:** 3-month outcomes by treatment assignment.

Outcome	PRIME2^a^ (*n* = 16)	Standard care^a^ (*n* = 18)	3-month difference	*P*^b^Value
Weight, lbs	− 3.4 (− 5.7, − 1.0)	0.4 (− 1.8, 2.5)	− 3.7 (− 6.8, − 0.7)	*0.02
Weight loss, %^c^	− 2.1 (− 3.7, − 0.4)	0.3 (− 0.9, 1.6)	− 2.4 (− 4.3, − 0.5)	*0.02
Body mass index, kg/m^2^	− 0.6 (− 1.1, − 0.2)	0.1 (− 0.4, 0.5)	− 0.7 (− 1.3, − 0.1)	*0.02
Waist circumference, cm	− 3.3 (− 5.4, − 1.2)	− 2.6 (− 5.3, 0.0)	− 0.6 (− 3.9, 2.7)	0.70
Hemoglobin A_1c_, %	− 0.3 (− 0.4, − 0.1)	− 0.1 (− 0.4, 0.2)	− 0.2 (-0.5, 0.1)	0.22
Systolic blood pressure, mmHg	0.0 (− 6.3, 6.3)	2.8 (− 2.0, 7.6)	− 2.8 (− 10.3, 4.7)	0.45
Diastolic blood pressure, mmHg	0.6 (− 3.4, 4.6)	− 0.1 (− 3.6, 3.5)	0.6 (− 4.5, 5.7)	0.81
Beck depression	0.1 (− 4.3, 4.5)	− 0.1 (− 3.4, 3.1)	0.2 (− 4.9, 5.4)	0.93
Cohen’s stress scale	− 0.4 (− 2.3, 1.6)	− 0.3 (− 2.5, 1.9)	− 0.1 (− 2.9, 2.8)	0.95

^a^ Observed 3-month differences in within-group outcomes were determined using paired *t*-test. ^**b**^*P* values correspond to independent *t* tests assessing significance of changes from baseline to 3 months between groups. ^c^ Percent weight loss is based on the weight loss from baseline to 3 months, determined using the following formula: [(3-month weight - baseline weight)/baseline weight x 100]. * that the P-value was less than 0.05.

**Table 3:** 6-month outcomes among PRIME2 participants.

Outcome	PRIME2^a^ (*n* = 12)	*P*^b^ Value
Weight, lbs	– 3.6 (– 9.0, 1.9)	*0.2
Body mass index, kg/m^2^	– 0.7 (– 1.8, 0.4)	*0.2
Waist circumference, cm	– 4.1 (– 7.6, – 0.6)	*0.0
Hemoglobin A_1c_, %	– 0.1 (– 0.3, 0.0)	0.1
Systolic blood pressure, mmHg	– 6.8 (– 14.0, 0.5)	0.1
Diastolic blood pressure, mmHg	– 0.6 (-5.0, 3.7)	1.0
Beck Depression	– 3.7 (– 11.6, 4.3)	0.5
Cohen’s Stress Scale	– 1.0 (– 3.3, 1.4)	0.5

^a^ Differences in within-group outcomes were determined using paired *t*-test. ^b^*P* values were derived from Wilcoxon signed-rank tests assessing significance of changes within the intervention group from baseline to 6 months. * that the P-value was less than 0.05.

## Results

**Fig. 2:**
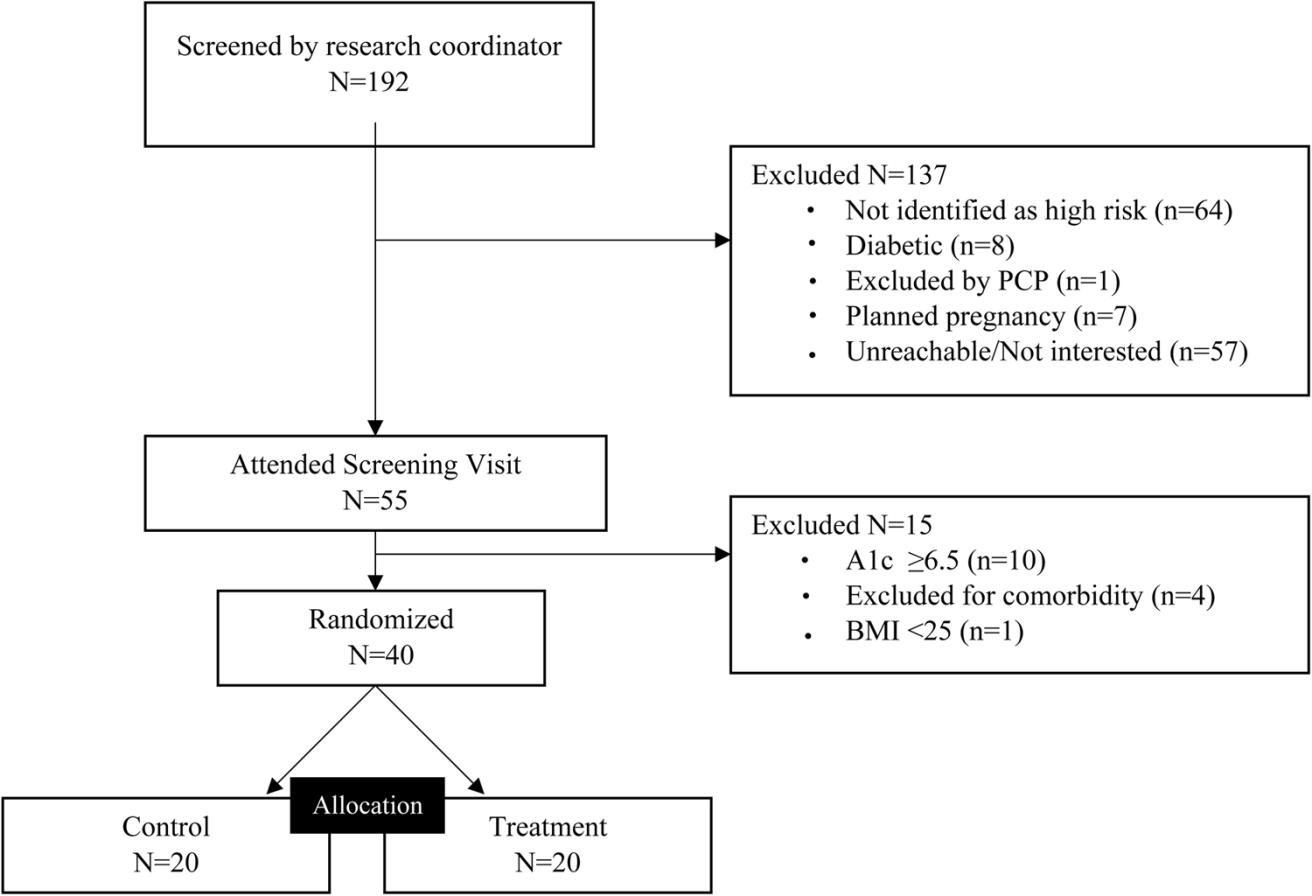
Flow of participants through the trial

Figure 2 displays the flow of participants through the study. In general, study participants were middle-aged Latinx women (95%), who were predominantly foreign born (90%), with limited educational attainment and mean household incomes below the federal poverty level. Overall, the mean weight was 179.7lbs (± 28.6), corresponding to a mean BMI of 33.3 kg/m^2^ (± 5.4), with a baseline HbA1c of 6.0% (± 0.2%). 75% of participants reported a family history of diabetes, and 12.5% had a personal history of gestational diabetes. At baseline, participants exhibited low to moderate levels of both perceived stress (18.8 ± 2.8) and depressive symptoms (11.3 ± 11.7). There were no significant baseline differences in any observed variables between the PRIME2 and enhanced usual care groups (Table 1).

PRIME2 participants attended an average of 10.1 (± 5.9) of the 16 sessions. Four participants (20%) in the PRIME2 group did not attend any lifestyle sessions, and 14 (70%) attended at least nine sessions.

Upon completion of the study interventions at 3 months, PRIME2 participants exhibited significant reductions in weight (− 3.7lbs, *p* = 0.02, 95% CI [− 6.8, − 0.7]); weight loss percent (− 2.4%, *p* = 0.02, 95% CI [− 4.3, − 0.5]); and BMI (− 0.7 kg/m^2^, *p* = 0.02, 95% CI [− 1.3, − 0.1]) relative to enhanced usual care. Within-group improvements in waist circumference and HbA1c among PRIME2 participants were not significantly different from those in the enhanced usual care group. There were no significant 3-month changes in perceived stress or depressive symptoms either within groups or between groups. (Table 2) Exploratory 6-month data from PRIME2 participants demonstrates that the weight loss observed during the 3-month period of active intervention was sustained. We found modest improvements in perceived stress and depressive symptoms at 6 months (− 1.0 and − 3.7, respectively). But these within-group differences from baseline were not statistically significant (Table 3).

## Discussion

This pilot trial demonstrates the feasibility and preliminary weight loss effectiveness of a promotora-led program integrating evidence-based behavioral approaches for healthy lifestyle change and stress reduction. For the co-primary outcomes at 3 months, we found significantly greater weight loss among PRIME2 participants relative to enhanced usual care, with no significant difference in perceived stress between groups. Our 6-month exploratory analysis of the PRIME2 group found durable reductions in weight, waist circumference, and systolic blood pressure, while showing small improvements in depressive symptoms and perceived stress that were greater than those observed at 3 months. High attendance and low rates of attrition from PRIME2 participants served as indicators of the PRIME2 intervention’s feasibility.

By focusing jointly on diabetes prevention and stress reduction, this study addresses an important comorbidity of metabolic and mental health. While many prior studies have established the association between stress and glycemic control, there has been little focus on developing and evaluating interventions aiming to improve both outcomes in an integrated way. Our intervention model employed promotoras, who are trusted community leaders with prior experience delivering similar interventions, as well as personal knowledge of local resources that could further support participants’ metabolic and mental health. The randomized design of this pilot trial represents another strength.

Our study has some notable weaknesses. We did not require participants to have elevated levels of perceived stress at baseline, given many sources of perceived stress that are prevalent in the target immigrant community. However, participants in the trial demonstrated a low to moderate level of baseline perceived stress according to their results on the Perceived Stress Scale, which may have limited the ability to demonstrate significant improvements in this construct. Further, stress levels were measured by participants’ self-report only, rather than objective physiologic measures such as salivary cortisol. A large proportion of our study sample (95%) were women, which limits the generalizability of study findings to this high-risk population. The small sample for this pilot study, and the short follow-up period for the primary outcomes, precluded a definitive assessment of the intervention’s effects.

Prediabetes and perceived stress are both common, impacting more than one-third of U.S. adults^1^. Because these related conditions share underlying pathophysiologic mechanisms and behavioral correlates, there is a unique opportunity to intervene upon them in an integrated way. To our knowledge, only one prior pilot study has addressed the comorbidity of prediabetes and psychological stress through an integrated intervention^34^. This 8-week intervention combined behavioral approaches from an effective mindfulness-based stress reduction program and a lifestyle change program developed by the National Diabetes Education Program. Among the 38 participants who received this intervention, there was a significant reduction in BMI (− 0.35 kg/m^2^; 95% CI [− 1.15, − 0.11]) at 3 months, which was no longer significant at 6 months (-0.45 kg/m^2^; 95% CI [− 1.14, 0.05]). This study also reported small but significant reductions in A1c at 3 months (− 0.08%; 95% CI [− 0.13, − 0.03]) and 6 months (− 0.12%; 95% CI [− 0.19, -0.06]). The participants in this pilot trial, who also exhibited low to moderate levels of baseline perceived stress, experienced small changes in perceived stress that were statistically significant at 3 months (-1.07; 95% CI [− 2.22, − 0.09]) but not at 6 months (− 0.27, 95% CI [− 1.48, 0.94]). This pilot trial also reported small changes in salivary cortisol levels that were not statistically significant. This trial reported a different study population, targeting African American participants with higher educational attainment and household income than those in the current study. These sociodemographic differences are factors that are known to impact both diabetes risk and trial outcomes^35^. However, this pilot intervention generally employed similar behavioral approaches to those used in the current study, while observing similar outcomes with respect to both metabolic and mental health endpoints.

Other pilot studies have developed and tested behavioral interventions for addressing obesity and stress, without requiring participants to have prediabetes. However, one of these combined materials from the Diabetes Prevention Program with several stress management techniques, including relaxation, behavioral approaches, and cognitive strategies^36^. This study of 44 participants, who exhibited much higher baseline levels of perceived stress than our participants (30.7 vs. 11.3), demonstrated similar percent weight loss from baseline (− 2.7% vs. − 2.1%) and a greater reduction in stress than those observed in our trial (− 5.3 vs. − 0.4)^36^. The comparable weight loss found in this study and ours likely relates to using the same intervention materials and intensity (16 weeks); whereas the larger stress reduction achieved in this earlier study may stem from higher levels of baseline stress found among their participants, who are more likely to derive benefit from such an intervention.

Another earlier trial tested a 10-week intervention teaching participants five stress reduction techniques, and addressing three specific lifestyle targets (i.e. 7–9 h of sleep/night, 30–60 min/day of exercise, and 60–90% of dietary intake from unprocessed foods)^37^. This intervention condition was compared with a group who participated in the Diabetes Prevention Program for 10 weeks. Participants in both intervention conditions experienced similar weight loss to those observed in our pilot trial, but greater reductions in perceived stress (i.e. − 5.2 for the stress reduction arm and − 2.4 for the Diabetes Prevention Program arm). The greater effectiveness of this prior study at lowering stress may also be related to higher levels of baseline perceived stress than those observed among our study participants (18.3 vs. 11.3). While the behavioral approaches for weight loss were similar across prior studies that used materials from the Diabetes Prevention Program, the stress reduction strategies may differ in ways that impacted their effectiveness. Future research should examine the comparative effectiveness of different evidence-based stress reduction techniques among participants with comorbid overweight/obesity and/or prediabetes.

### Implications for practice and research

This work supports the continuing body of DPP translational research with implications for community-based practice. Our findings demonstrate modest weight loss is achievable at shorter program durations than the full year-long DPP. Although many studies have translated the DPP into “real world” settings, none have measured its effectiveness utilizing a promotora-led intervention spanning both metabolic health and mental health content. The novel PRIME2 intervention demonstrates that promotoras can deliver effective and culturally affirming group interventions focused on the intersection of metabolic and mental health.

## Conclusions

In conclusion, this pilot study developed and tested a combined intervention addressing prediabetes and stress together, with metabolic and mental health outcomes that were comparable with the only prior study targeting these comorbid conditions. Prior interventions focused on the behavioral management of obesity and stress found similar weight loss and greater stress reduction than that reported here. To our knowledge, all studies in this area have been pilot trials including less than 50 participants. Prior to widespread implementation of such programs in the field, future research should include larger randomized samples that enable a definitive evaluation of intervention effects on both metabolic and mental health endpoints. A large body of prior research has focused on the intersection of depression and obesity, elucidating complex relationships between intervention approaches and outcomes in this comorbid population. Future research combining behavioral management of stress with obesity and/or prediabetes should develop best practices for identifying target populations that are most likely to benefit and intervention approaches that maximize improvements in these metabolic and mental health outcomes.

## Electronic Supplementary Material

Below is the link to the electronic supplementary material.

Supplementary Material 1.

## Data Availability

The datasets generated during and/or analyzed during the current study are available from the corresponding author on reasonable request.
