# Supplementary material for: Promotora intervention for metabolic and mental health to reduce type 2 diabetes risk: a pilot randomized controlled trial
Source: Sci Rep. 2024 Dec 30;14:31988. doi: 10.1038/s41598-024-83482-5 (PMC11686005; doi:10.1038/s41598-024-83482-5)
Supplement: Supplementary file 1 — Supplementary Material 1. [file 41598_2024_83482_MOESM1_ESM.docx]

**Supplementary Table S1: Integration of DPP with Cognitive Behavioral Therapy content**CBT = Cognitive behavioral therapy

| **DPP content** | **CBT content** | **Integration** |
| --- | --- | --- |
| 1. Introduction to DPP (part 1) |  |  |
| 2. Introduction to DPP (part 2) | a. Awareness of thoughts/emotions | 2 + a |
| 3. Be a fat and calorie detective (part 1) | b. Recognizing emotions | 3 + b |
| 4. Be a fat and calorie detective (part 2) | c. Recognizing physical sensations | 4 + c |
| 5. Healthy eating (part 1) | d. Triggers for emotions | 5 + d |
| 6. Healthy eating (part 2) | e. Information processing | 6 + e |
| 7. Move those muscles (part 1) | f. Distorted thinking | 7 + f, g |
| 8. Move those muscles (part 2) | g. Cognitive restructuring | 8 + g |
| 9. Tip the calorie balance |  | 9 + g |
| 10. Take charge of what’s around you | h. Rational thought replacement | 10 + g, h |
| 11. Problem solving | i. Anger management | 11 + h, i |
| 12. Stay Active |  | 12 + c |
| 13. The slippery slope of lifestyle change |  | 13 + f, g |
| 14. Four Keys to Healthy Eating Out | j. Communication skills | 14 + j |
| 15. Make social cues work |  | 15 + a, e, g |
| 16. Ways to stay motivated |  | 16 + h, j |
